# Quality of pediatric trauma care: development of an age-adjusted TRISS model and survival benchmarking in a major trauma center

**DOI:** 10.3389/fped.2024.1481467

**Published:** 2024-12-12

**Authors:** Ana De los Ríos-Pérez, Alberto Federico García, Paula Gomez, Juan José Arias, Andrés Fandiño-Losada

**Affiliations:** ^1^ Pediatric Emergency Medicine, Fundación Valle del Lili Teaching Hospital, Cali, Colombia; ^2^ Faculty of Health Sciences, Universidad Icesi, Cali, Colombia; ^3^ Trauma & Acute Surgery, Critical Care, Fundación Valle del Lili Teaching Hospital, Cali, Colombia; ^4^ Faculty of Health, Universidad del Valle, Cali, Colombia; ^5^ Cisalva Institute, Faculty of Health, Universidad del Valle, Cali, Colombia

**Keywords:** injuries, pediatric trauma, trauma severity indices, trauma score, TRISS, trauma injury severity score, survival prediction, quality of care

## Abstract

**Background:**

Pediatric trauma is a major global health concern, accounting for a substantial proportion of deaths and disease burden from age 5 onwards. Effective triage and management are essential in pediatric trauma care, and prediction models such as the Trauma Injury Severity Score (TRISS) play a crucial role in estimating survival probability and guiding quality improvement. However, TRISS does not account for age-specific factors in pediatric populations, limiting its applicability to younger patients. This study aimed to modify TRISS to account for age for children (Peds-TRISS) and to evaluate its performance relative to the original TRISS. We also assessed survival outcomes to explore the model's potential utility across various clinical settings. These efforts align with quality improvement initiatives to reduce preventable mortality and supporting sustainable development goals.

**Methods:**

This retrospective cohort study included patients under 18 years of age who were treated at a hospital in Colombia between 2011 and 2019. New coefficients for TRISS covariates were calculated using logistic regression, with age treated as a continuous variable. Model performance was evaluated based on discrimination (C statistic) and calibration, comparing Peds-TRISS with the original TRISS. Internal validation was conducted using bootstrap resampling. Survival outcomes were assessed using the M and Z statistics, which are commonly used for international trauma outcome comparisons.

**Results:**

The study included 1,013 pediatric patients with a median age of 12 years (IQR 5–15), of whom 73% were male. The leading causes of injury were traffic accidents (31.1%), falls (28.8%), and assaults (28.7%). The overall mortality rate was 5.7%. The Peds-TRISS model demonstrated good calibration (HL = 9.7, *p* = 0.3) and discrimination (C statistic = 0.98, 95% CI 0.97–0.99), with no statistically significant difference in the ROC curve comparison with the original TRISS. Internal validation demonstrated strong performance of Peds-TRISS. The M and *Z* statistics were 0.93 and 0, respectively, indicating no significant differences between expected and observed survival rates.

**Conclusions:**

Most fatalities occurred among adolescents and were due to intentional injuries. The Peds-TRISS model showed a partial improvement in performance compared to the original TRISS, with superior results in terms of calibration, although not in discrimination. These findings highlight the potential of model customization for specific populations. Prospective, multicenter studies are recommended to further validate the model's utility across diverse settings.

## 1 Introduction

Injuries among children and adolescents represent a significant global health challenge, with more than 1,600 individuals under the age of nineteen dying daily due to trauma. This burden is particularly pronounced in low- and middle-income countries, where both the incidence and impact of injuries exceed those observed in high-income regions (1, 2). Among individuals aged 5–29, 3 of the 5 leading causes of death are injury-related, including traffic accidents, falls, homicides, and suicides (3). While unintentional injuries account for the majority of these deaths globally (4), Colombia presents a distinct trauma epidemiology. In this country, interpersonal violence emerges as the leading cause of death starting at age 10 and ranks as the fourth leading cause from age 5 onward (5).

Both the World Health Organization (WHO) (6) and the Centers for Disease Control and Prevention (CDC) (7) recognize injuries as the number one killer of young people, underscoring the urgent need for effective strategies to mitigate the toll of trauma on this vulnerable population. Beyond fatalities, tens of millions of individuals worldwide suffer non-fatal injuries each year, resulting in long-term healthcare needs, rehabilitation, and substantial economic impacts due to premature death and disability (8–10).

To address this burden, international trauma care authorities emphasize strategies to improve trauma care quality and outcomes. Survival prediction models play a central role in these efforts, providing tools to estimate survival probabilities and guide clinical decision-making (11). However, while numerous prediction tools have been developed and validated for adult trauma populations, similar tools specifically tailored to pediatric trauma remain scarce. This gap is particularly concerning, given the distinct physiological and epidemiological characteristics of pediatric patients, which differ significantly from those of adults. Accurate predictive models for pediatric trauma are essential to guide clinical decision-making, improve outcomes, and address the unique needs of children.

The Trauma and Injury Severity Score (TRISS) is one of the most widely used tools in trauma centers for assessing patient survival likelihood, enabling healthcare providers to identify high-risk patients and prioritize care (12–16). Additionally, TRISS supports trauma center quality improvement by comparing observed vs. expected outcomes, facilitating benchmarking, and identifying gaps in care (17, 18). This approach aligns with the WHO's Guidelines for Trauma Quality Improvement Programmes, which advocate for strategies to reduce the burden of trauma worldwide (19).

However, TRISS was originally developed for adult populations, and its predictive accuracy is limited in pediatric patients (20, 21). This limitation arises from the model's use of age as a binary variable, which overlooks the nuanced impact of age on survival in children (22, 23). Studies have shown that modifying TRISS to treat age as a continuous variable or recalculating its coefficients can significantly enhance its predictive power for pediatric populations (24–26).

Despite these advancements, pediatric-specific adaptations of TRISS remain limited, as most survival prediction models have been optimized for adults rather than children. This issue is particularly relevant in Colombia, where the unique trauma epidemiology—characterized by high rates of violence and other trauma-related causes—emphasizes the need to test and adapt predictive tools for pediatric populations in Latin America.

In this study, we aimed to adapt TRISS to account for pediatric age as a continuous variable (Peds-TRISS) and evaluate its performance among children treated at our trauma center in Colombia. Rather than creating a new model, we focused on refining the existing TRISS framework to improve its applicability to pediatric trauma, ultimately contributing to evidence to improve quality of care and outcomes in this vulnerable population.

## 2 Materials and methods

### 2.1 Study design and setting

A retrospective study was conducted in the emergency department at Fundación Valle del Lili Hospital in Cali, Colombia, between January 2011 and May 2019. Cali is served by three major trauma centers, including our hospital. Our institution operates as a Level I trauma center with 721 beds, including 250 dedicated to critical care. It handles approximately 8,000 trauma cases annually, of which 1,000 involve severely injured patients, and 700 require trauma code activation. In addition to trauma care, the hospital manages other complex pathologies and serves as a referral center for the southwestern region of the country.

### 2.2 Participants

Patients under 18 years old who were treated in the emergency department for trauma injuries (discharge diagnosis codes between S00 and T149, according to the International Classification of Diseases, 10th edition) were included in the review. Patients were selected if they required hospitalization, had a hospital stay longer than 6 h, or died within this period. Excluded from the study were those presenting with injuries related to drowning, burns, foreign bodies, poisoning, medical-surgical complications, or sequelae from prior trauma.

Additional exclusion criteria included patients transferred from regions outside Valle del Cauca or Cauca, those presenting more than 24 h after the trauma, and those with a history of oncological, hematological, metabolic, or osteoarticular diseases that might affect their treatment or prognosis. Patients transferred from other hospitals where they had already undergone surgery were also excluded, as this precluded accurate classification of their initial injuries and severity. Furthermore, patients transferred to another hospital before the 30th day of hospitalization were excluded due to an inability to verify their vital status at that time.

### 2.3 Data sources and measurement

Patient data were sourced from electronic medical records, ensuring comprehensive access to all clinical and administrative information. The chart review process involved a combination of systematic data extraction for structured fields and manual review for unstructured data. Sociodemographic variables, trauma mechanisms, causes of trauma, and injury severity were collected. Clinical variables at admission, including heart rate, respiratory rate, blood pressure, and Glasgow Coma Scale scores, were also recorded. Radiology and surgery reports were thoroughly reviewed to ensure accuracy and completeness. The primary outcome variable was mortality within 30 days of hospitalization or earlier if the patient died or was discharged home.

Information such as diagnostic codes, radiology findings, and surgery reports was manually verified to ensure completeness and accuracy. Quality control measures were applied throughout the data collection process. A random 15% sample of the collected data was subjected to a double-checking process, where two independent investigators cross-verified and reconciled discrepancies.

### 2.4 Trauma injury severity score (TRISS)

TRISS is a score that combines physiological and anatomical variables, and is used to estimate the probability of patient survival after in trauma. For its calculation, two scores (Revised Trauma Score-RTS and Injury Severity Score-ISS), the mechanism of injury (blunt or penetrating), and age are considered. The formula is:

$$\;\;\;\;\mathrm{Ps}=\frac{1}{1\;+\;e^{-b}}$$

Where: Ps = probability of survival, *e* is a constant (approximately 2.718282, the base of the natural or Napierian logarithm), b = b0 + (b1xRTS) + (b2xISS) + (b3xage). Here, b0, b1, b2, and b3 are coefficients that differ according to the mechanism of the lesion, that is, if it is blunt or penetrating, and is derived from a logistic regression model based on data from the Major Trauma Outcome Study (MTOS) in North America (22, 23). The age variable is dichotomized with its coefficient being zero for those under 55 years of age, making it null in the equation for the pediatric population. For this reason, coefficients for RTS, ISS, and age were recalculated in our study, considering age as a continuous variable and accounting for the blunt or penetrating mechanism in the development of the multivariable logistic regression model, which has not been evaluated in pediatric patients in our region. The TRISS result (Ps) ranges from 0 to 1 (23) and was calculated with the new coefficients for included patients.

### 2.5 Revised trauma score (RTS), abbreviated injury scale (AIS) and injury severity score (ISS)

RTS accounts for three physiological variables in its formula: Glasgow Coma Scale, blood pressure, and respiratory rate, and its value ranges between 0 and 7.84 (27). The AIS serves as the basis for calculating the ISS, classifying injuries across six body regions (28). Various versions of this score have been developed; we used the 2015 version (29). The ISS is determined by summing the squares of the AIS scores for the three most severely injured regions, yielding a score between 1 and 75. An ISS of 16 or higher indicates a serious injury (30–32).

### 2.6 Statistical methods

Categorical variables were presented as frequencies and proportions, and comparisons between survivors and non-survivors were conducted using the chi-square test with Yates' correction for continuity. Continuous variables were presented as median and interquartile range (IQR), and comparisons between survivors and non-survivors were performed using Wilcoxon-Mann-Whitney Test. For the development of the new model, the coefficients of the RTS, ISS and Age variables were calculated using a logistic regression model, considering age as a continuous quantitative variable and the discharge status (alive/dead) as the outcome variable. The TRISS was calculated using the new coefficients (Peds-TRISS). Its performance in predicting survival was evaluated through discrimination and calibration. Discrimination assesses how well the model distinguishes between individuals who do and do not develop the outcome of interest. It was measured using the area under the receiver operating characteristic curve (AUROC), where a value of 1 indicates perfect discrimination and 0.5 indicates no better than chance. The AUROC of Peds-TRISS and original TRISS was compared using the DeLong test and the *p* < 0.05 was regarded as significantly different. Calibration, which assesses the degree of agreement between predicted and observed probabilities, was measured using the Hosmer-Lemeshow goodness-of-fit test, where a *p*-value greater than 0.05 indicates good calibration (33). We compared the Peds-TRISS and original TRISS, using the Akaike Information Criterion (AIC) and the Bayesian Information Criterion (BIC), where lower values indicate a better model (34, 35).

The internal validation was carried out following the Transparent Reporting of a multivariable prediction model for Individual Prognosis or Diagnosis (TRIPOD) recommendations (36). Bootstrapping resampling with 500 resamples was used, a powerful and efficient technique that provides more stable and less biased estimates of the model's performance and is widely recognized in predictive model research (36–40). The performance measures from the validation were discrimination with the C-statistic and calibration measured with calibration-in-the-large (CITL, ideal value of 0), calibration slope (ideal value of 1) and observed: expected ratio (E:O ratio, ideal value of 1) (33, 39).

### 2.7 Benchmarking survival assessment

Among the outcome evaluation and quality assurance tools are the DEF (DEFinitive outcome-based evaluation) statistical methods. These include the Z and W statistics, internationally used to compare trauma survival or mortality rates between two institutions (23, 41–43). The Z statistic, initially described by Flora in 1978 (44), compares observed survival in a group of patients with predicted survival according to the reference group (MTOS) and quantifies the difference (23). The formula is:

$$\;Z\;=\;\frac{S-\sum \mathrm{Pi}}{\surd \left(\sum \mathrm{PiQi}\right)}\;$$

Where: S = observed survivors, ΣPi = is the sum of expected survival probabilities, Qi = probability of death (1-Pi), ΣPiQi = sum of the product of survival and death probabilities (23). A Z value between −1.96 and +1.96 is not statistically significant, meaning there is no evidence that the evaluated group has a survival rate significantly different from the reference with a significance level of 0.05 (23, 45). For the statistical process to be valid, Pi and Qi must be at least 5 (45). Since the *Z* value can be affected by differences in the severity of injuries between comparison groups, the M statistic measures comparability between groups. To calculate it, the survival probability is divided into 6 ranges, and the fraction of patients in each range is compared between both groups, quantifying their differences. Its formula is: M = S1 + S2 + S3 + S4 + S5 + S6, where S is the minimum value between both groups in each range. A value of M between 0.88 and 1 indicates that both groups are similar (23).

The sample size was calculated using a proportional comparison formula. *A priori*, the estimated sample size was 927 children, calculated to detect a 2% difference between observed and expected mortality.

The statistical analysis was performed using Stata version 17 (StataCorp, College Station, Texas 77845, USA).

### 2.8 Ethical considerations

This study was conducted in accordance with the Declaration of Helsinki and was approved by the Biomedical Research Ethics Committee of the hospital under registration number 295-2018. Given the retrospective observational nature of the study, which involved the anonymous collection of systematically gathered data, informed consent was not required, as per the decision of the ethics committee and Colombian legislation.

## 3 Results

**Figure 1:**
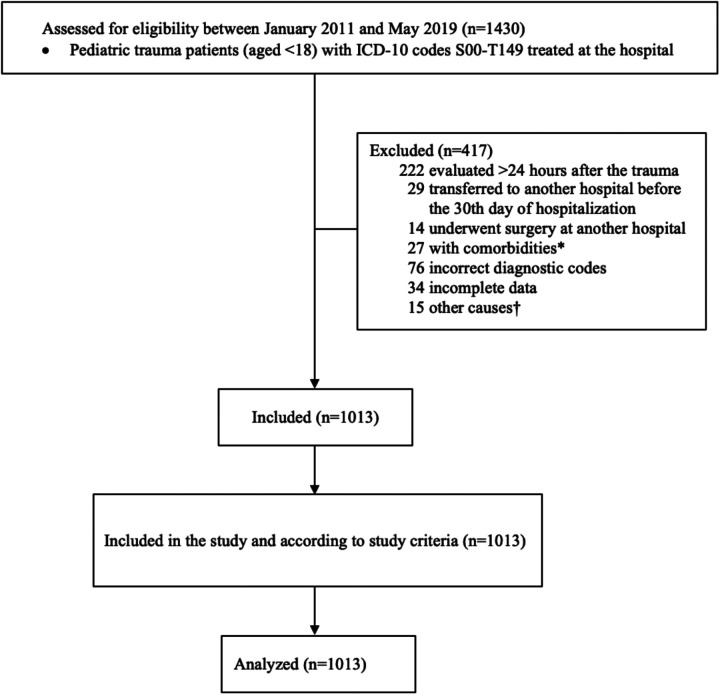
Flow chart showing the inclusion and exclusion criteria for study population. ICD-10, International classification of diseases, 10th edition. *Comorbidities: oncological, hematological, metabolic or osteoarticular comorbidities. ^†^Other causes: unknown time since trauma, escape, transfer from provinces other than Valle del Cauca or Cauca.

**Figure 2:**
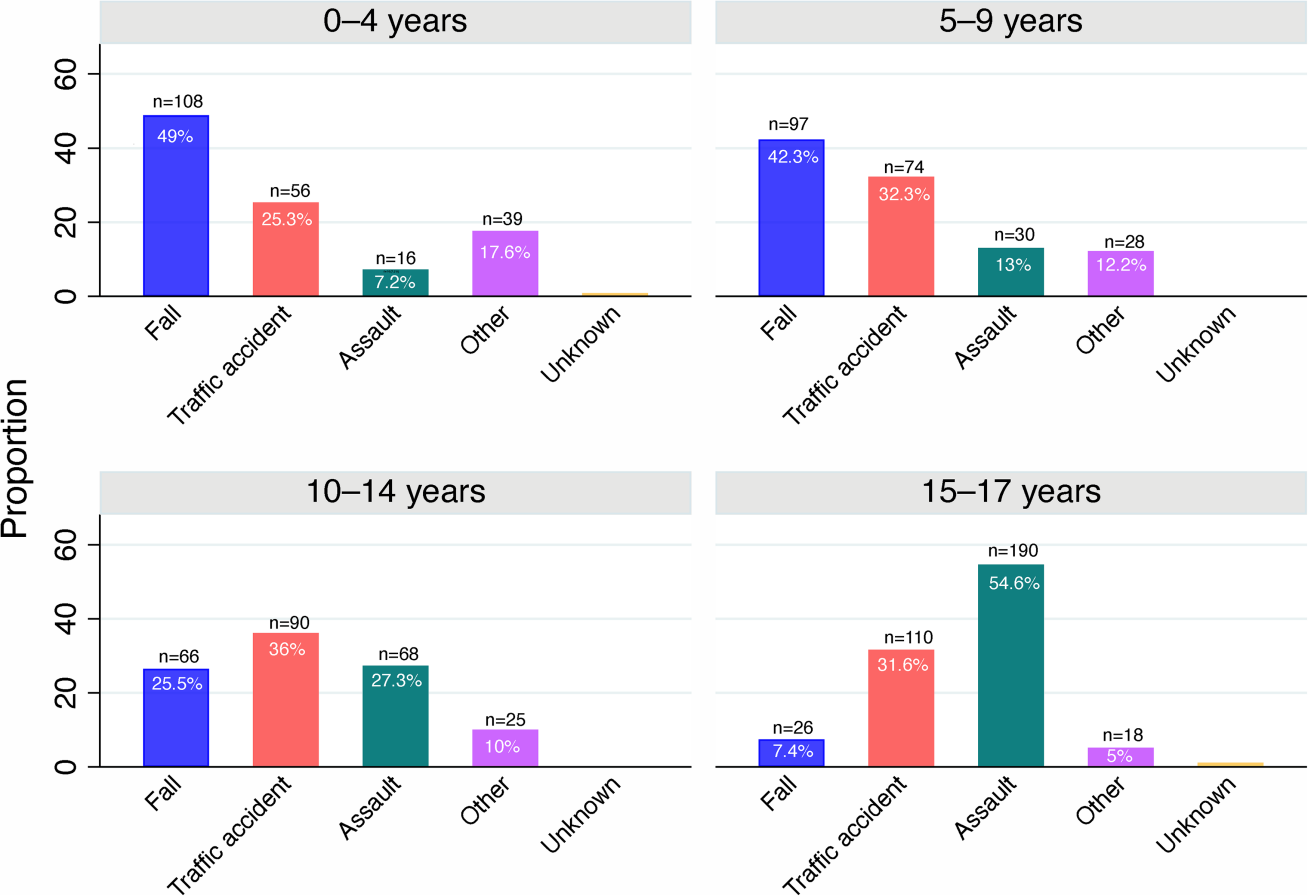
Cause of trauma by age group.

A total of 1,013 children were enrolled in the study, with a complete data analysis performed after excluding 34 patients due to missing Glasgow Coma Scale information (Figure 1). The overall percentage of missing data was low (3.2%), and no imputation was conducted. Sociodemographic and clinical characteristics are summarized in Table 1 and were published in another article with a slightly larger sample that did not require the exclusion of the mentioned patients (46). The median age of participants was 12 years (IQR, 5–15 years), 73% being male. Age distribution was as follows: 21.2% were aged 0–4 years, 21.8% were aged 5–9 years, 23.9% were aged 10–14 years, and 33.1% were aged 15–17 years. Most patients (66.2%) sustained blunt trauma, while 33.8% experienced penetrating trauma. The subsidized health insurance system provided coverage for 32.9% of the patients, whereas 67.1% were insured under the contributory system, which includes private insurance. The leading causes of injury were traffic accidents (31.1%), falls (28.8%), and assaults (28.7%) (Figure 2), with firearms responsible for 97% of the violence-related deaths. Thirty-two percent of patients presented with severe trauma (ISS ≥16).

**Table 1:** Sociodemographic and clinical characteristics.

	Overall (*n* = 1,013)	Alive (*n* = 955)	Dead (*n* = 58)	*p* value
Median age, years (IQR)	12 (5–15)	11 (5–15)	15 (13–17)	<0.001
Age groups				<0.001
0–4 years	215 (21.2%)	209 (21.9%)	6 (10.3%)	
5–9 years	221 (21.8%)	217 (22.7%)	4 (6.9%)	
10–14 years	242 (23.9%)	230 (24.1%)	12 (20.7%)	
15–17 years	335 (33.1%)	299 (31.3%)	36 (62.1%)	
Sex, *n* (%)				0.062
Male	740 (73%)	691 (72.4%)	49 (84.5%)	
Female	273 (27%)	264 (27.6%)	9 (15.5%)	
Payor status, *n* (%)				<0.001
Private insurance	680 (67.1%)	657 (68.8%)	23 (39.7%)	
Subsidized	333 (32.9%)	298 (31.2%)	35 (60.3%)	
Origin, *n* (%)				0.159
Valle	752 (74%)	714 (74.8%)	38 (65.5%)	
Cauca	261 (26%)	241 (25.2%)	20 (34.5%)	
Transferred from another hospital^a^	693 (69.2%)	644 (68.2%)	49 (86%)	0.005
Mechanism of injury				<0.001
Blunt	671 (66.2%)	647 (67.7%)	24 (41.4%)	
Penetrating	342 (33.8%)	308 (32.3%)	34 (58.6%)	
Age-adjusted hypotension	72 (7%)	57 (6%)	15 (26%)	<0.001
Glasgow Coma Scale (IQR)	15 (13–15)	15 (14–15)	3 (3–6)	<0.001
ISS, median (IQR)	9 (5–17)	9 (4–16)	31 (25–37)	<0.001
RTS, median (IQR)	7.84 (7.11–7.84)	7.8 (7.5–7.8)	4 (4–5)	<0.001
TRISS, median (IQR)	0.99 (0.98–0.99)	0.99 (0.99–0.99)	0.51 (0.2–0.66)	<0.001
Peds-TRISS, median (IQR)	0.99 (0.99–0.99)	0.99 (0.99–0.99)	0.33 (0.13–0.48)	<0.001
ICU				<0.001
Yes	490 (48.4%)	443 (46.4%)	47 (81%)	
No	523 (51.6%)	512 (53.6%)	11 (19%)	
Transfusion				<0.001
Yes	251 (24.8%)	221 (23.1%)	30 (51.7%)	
No	762 (75.2%)	734 (76.9%)	28 (48.3%)	
Surgery				0.062
Yes	632 (62.4%)	603 (63.1%)	29 (50%)	
No	381 (37.6%)	352 (36.9%)	29 (50%)	
Hospital stay, hours median (IQR)	3.5 (1.7–6.9)	86.4 (43.2–168)	21.6 (4.8–50.4)	<0.001

Data are *n* (%) or median (IQR). IQR, interquartile range; ISS, injury severity score; RTS, revised trauma score; TRISS, trauma injury severity score; ICU, intensive care unit.

^a^ Data available for 1,002 patients.

A bivariate analysis of characteristics by survival status at discharge is presented in Table 1. The median age of deceased patients was 15 years (IQR, 13–17 years), compared to 11 years (IQR, 5–15 years) in survivors (*p* < 0.001). Most deaths (83%) occurred among patients aged 10–17 years, with males representing 90% of this group. Among the deceased, 60.3% were covered by subsidized health insurance. Violence was the leading cause of death, followed by traffic accidents (61.3% and 33.8%, respectively). The median ISS in deceased patients was 31 (IQR, 25–37), compared to a median ISS of 9 (IQR, 4–16) in survivors. The most frequent fatal injury was traumatic brain injury (91.3%). The overall mortality rate was 5.7%, with deaths occurring at a median of 15 h post-injury (IQR, 3.8–49 h), and no deaths occurred beyond 20 days of hospitalization.

**Figure 3:**
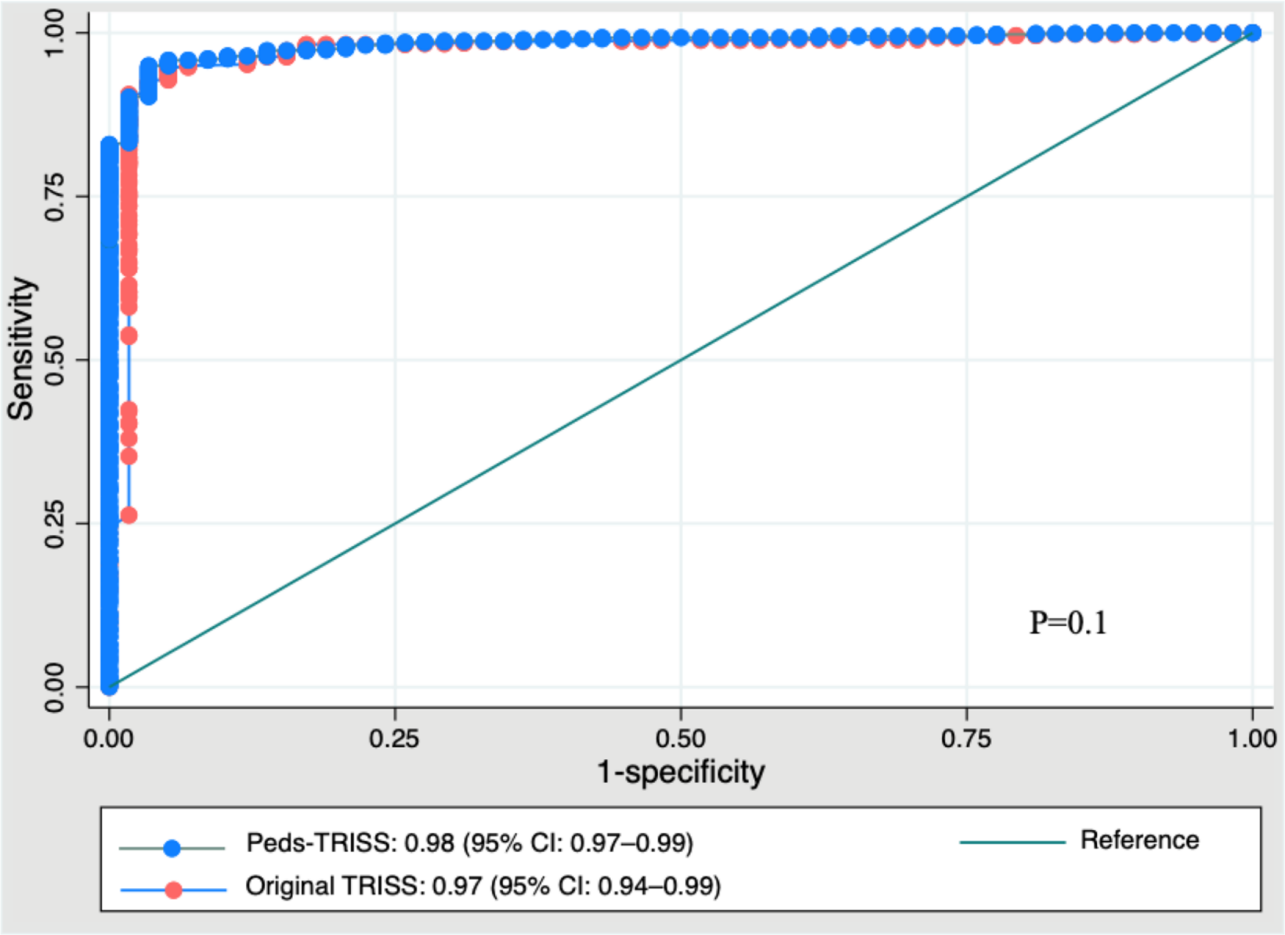
Comparative AUROC of Peds-TRISS and original TRISS. AUROC, area under the receiver-operating characteristic curve; TRISS, trauma injury severity score; CI, confidence interval.

The coefficients of the Peds-TRISS are presented in Table 2. In terms of performance, a comparison between the Peds-TRISS and the original TRISS was conducted. Both demonstrated strong discrimination (Table 3). The area under the receiver-operating characteristic curve (AUROC) was 0.971 (95% CI, 0.945–0.996) for the original TRISS and 0.984 (95% CI, 0.961–0.993) for the Peds-TRISS. The DeLong test revealed no statistically significant difference in AUROCs (*p* > 0.05) (Figure 3). However, calibration was satisfactory only in the Peds-TRISS model, as indicated by the Hosmer–Lemeshow statistic (HL = 9.7, *p* = 0.3 for the Peds-TRISS and HL = 16.6, *p* = 0.03 for the original TRISS). Additionally, the Akaike Information Criterion (AIC) and Bayesian Information Criterion (BIC) were lower for the Peds-TRISS (AIC = 187, BIC = 197) compared to the original TRISS (AIC = 228, BIC = 238), supporting its selection based on model fit. An additional analysis of Peds-TRISS performance by age subgroup was conducted, demonstrating robust performance of the score across all age groups, as measured by discrimination and calibration (Table 4).

**Table 2:** Coefficients of the Peds-TRISS model derived from the study database.

	Blunt trauma		Penetrating trauma	
	Coefficient	Standard error	Coefficient	Standard error
b0	2.406	1.100	4.293	1.833
b1 (RTS)	0.744	0.161	0.811	0.134
b2 (ISS)	−0.137	0.030	−0.121	0.039
b3 (Age)	0.073	0.058	−0.190	0.096

TRISS, trauma injury severity score; RTS, revised trauma score; ISS, injury severity score.

**Table 3:** Comparative performance of TRISS models.

	AUROC (95% CI)	HL	AIC	BIC
TRISS	0.971 (0.945–0.996)	16.6 (*p* = 0.03)	228	238
Peds-TRISS	0.984 (0.961–0.993)	9.7 (*p* = 0.3)	187	197

*N*, 1,013; TRISS, trauma injury severity score; AUROC, area under the receiver-operating characteristic curve; CI, confidence interval; HL, Hosmer-Lemeshow; AIC, Akaike information criterion; BIC, Bayesian information criterion.

**Table 4:** Peds-TRISS performance by age group.

	*n*	AUROC (95% CI)	HL (*p*)
Peds-TRISS (all ages)	1,013	0.9841 (0.9758–0.9924)	9.69 (0.3)
<5 years	215	0.9928 (0.9827–1)	1.02 (0.9981)
5–9 years	221	0.9862 (0.9670–1)	1.45 (0.9935)
10–14 years	242	0.9891 (0.9776–1)	2.01 (0.9808)
15–17 years	335	0.9690 (0.9484–0.9894)	8.33 (0.4023)

Peds-TRISS, age-adjusted trauma injury severity score; AUROC, area under the receiver-operating characteristic curve; HL, Hosmer-Lemeshow.

**Figure 4:**
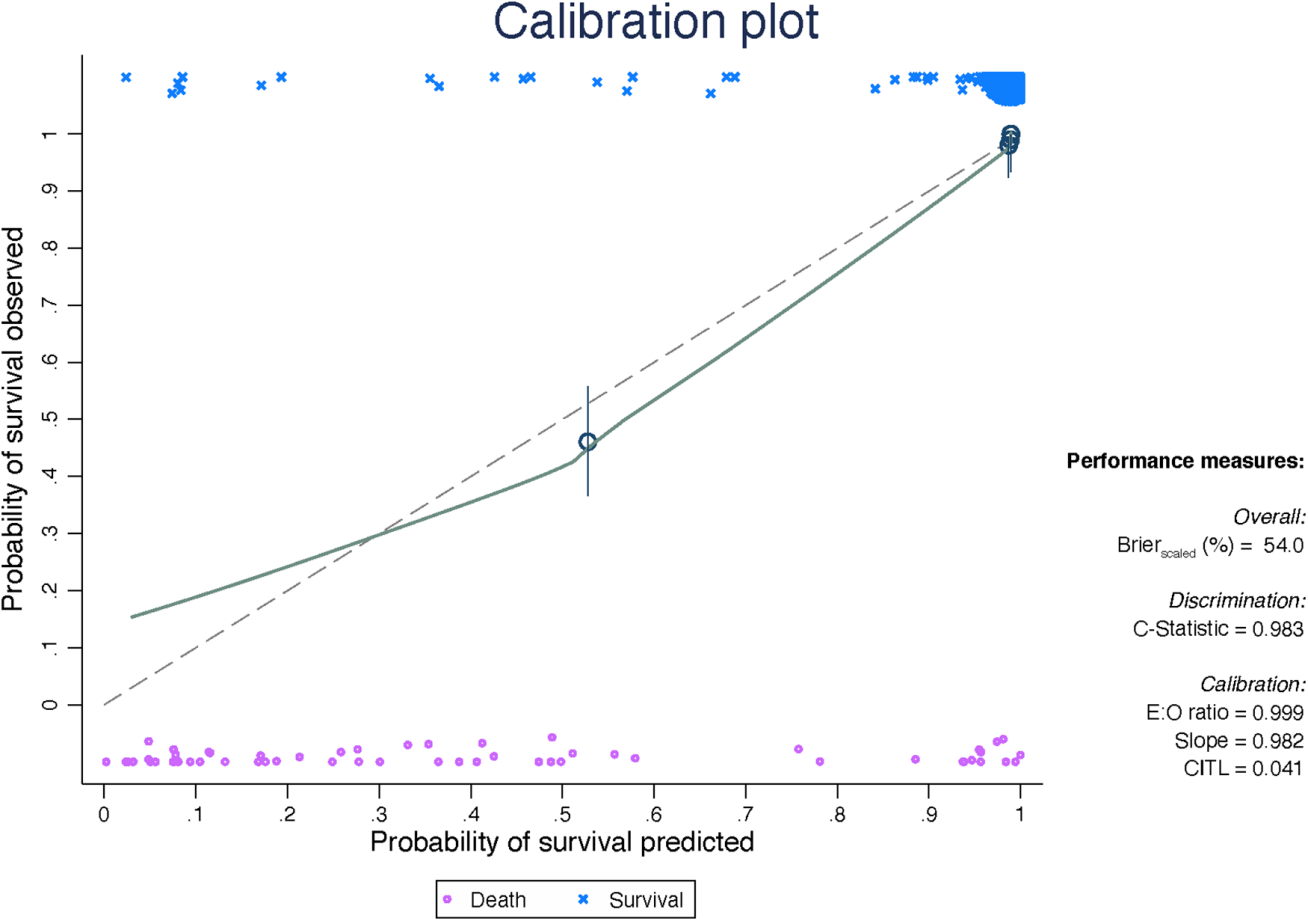
Internal validation of the peds-TRISS. E:O ratio = ratio between expected and observed events. CITL, calibration-in-the-large.

The internal validation of the Peds-TRISS demonstrated strong performance across multiple metrics, including discrimination, calibration, and model fit, as shown in Figure 4.

For the benchmarking assessment, the M statistic was 0.93, and the Z statistic was 0, indicating no statistically significant difference in survival rates between our patient cohort and the expected outcomes as defined by the comparison standard.

## 4 Discussion

This study assessed the performance of Peds-TRISS, an age-adjusted TRISS-based survival prediction model in a pediatric population in Colombia, using trauma center data from patients under 18 years old collected between 2011 and 2019. A significant proportion of deaths within this cohort were due to violent trauma mechanisms, and the Peds-TRISS performance was compared with that of the original TRISS. Our findings differ from the global literature, which predominantly reports trauma-related deaths due to unintentional injuries (4, 8), but align with studies conducted in Latin America (47, 48). For example, research from Brazil highlights that intentional trauma, particularly homicides, plays a significant role in adolescent mortality (49). Similarly, data from Mexico identify assaults as a major cause of trauma among adolescents, emphasizing the influence of violence on mortality patterns (50). Consistent with these findings, the SALURBAL study reports that homicides among adolescents and young adults represent a serious public health problem in Latin America, reinforcing the region's designation as one of the most violent in the world (44).

In Colombia, injuries constitute the first three causes of death from age 10, with homicide leading the list (5). This reflects a public health problem of significant magnitude, requiring substantial investment of human, financial, technical, and technological resources across the continuum of care—from pre-hospital services to long-term rehabilitation. Additionally, the emotional, social, and economic implications are profound, particularly due to the loss of productive years through premature death or disability (51–53). This shared regional burden underscores the need for effective interventions and predictive tools that are tailored to the specific epidemiological patterns observed in low- and middle-income countries (LMICs).

The implementation of survival prediction tools in pediatric trauma is strongly recommended by international authorities, such as the American College of Surgeons Committee on Trauma (ACS COT) in Advanced Trauma Life Support and the National Institute for Health and Care Excellence (NICE) in the United Kingdom (54, 55). These organizations recognize the utility of applying these scores for effective trauma system functioning. One of the most commonly used survival prediction tools in trauma research is TRISS, which has critical implications for patient management and trauma center quality assessment (12–16, 20) but has been less evaluated and documented in pediatric patients. TRISS incorporates anatomical and physiological variables, with the latter playing a vital role in children's adaptive response to trauma (30, 56–58). However, the parameters used (respiratory rate, systolic blood pressure) are based on findings from adult populations, limiting their reliability in pediatric settings, as these values vary significantly with age.

Our evaluation of the original TRISS model in our pediatric cohort of patients revealed limitations in its performance. Previous studies have identified similar constraints, noting increased predictive power with modifications to variables like age, such as expanding age group categorizations, recalculating coefficients, or treating age as a continuous quantitative variable rather than a binary category (20, 24–26). Despite these limitations, these studies acknowledge TRISS's utility as a tool for evaluating trauma care quality improvement systems, making it the most widely used scale in injury survival studies. Additionally, a systematic review of trauma mortality prediction models underscores the importance of including demographic predictors, ideally quantitative, to enhance research quality and model performance (11).

Consistent with previous studies, we aimed to enhance the TRISS model by incorporating age as a continuous variable and recalculating all model coefficients. Our analysis revealed that the ROC curve for the Peds-TRISS model was nearly identical to that of the original TRISS, indicating that the age adjustment contributes minimally and without statistical significance to its discriminatory power. However, the adjusted model showed superior performance in terms of calibration, suggesting that the age-adjusted version aligns predicted and observed outcomes more accurately across all risk levels. This underscores the potential of tailored modifications to refine predictive models for specific populations. Furthermore, earlier studies have reported improvements in model performance following adjustments to variables such as age (20, 24, 25), highlighting the value of these modifications in enhancing predictive tools for specific clinical and epidemiological contexts.

The development and refinement of models like Peds-TRISS emphasize the importance of leveraging innovative tools to enhance trauma care, particularly in regions facing significant public health challenges. Recent advancements in artificial intelligence (AI) provide a promising avenue for further improving trauma prediction models, enabling real-time, personalized outcome predictions. By integrating large-scale data—such as imaging, vital signs, and patient history—AI-driven systems could optimize triage, refine risk stratification, and support clinical decision-making. However, for AI to achieve its full potential in pediatric trauma, it is crucial to adapt these technologies to the unique physiological characteristics of children and ensure access to high-quality data. Future research integrating AI into models like Peds-TRISS could facilitate more precise and actionable insights for trauma care in diverse clinical settings (59).

In addition to refining predictive models, comparative evaluation of patient outcomes provides a valuable measure of trauma center care quality (17–19). Comparative statistics enable the assessment of observed survival rates for injured patients at a trauma center against expected survival rates from national or international reference centers. The literature reports varied results (41–43); however, our findings showed no statistically significant differences between our patients and the MTOS group (*Z* = 0), with statistically valid comparability between both groups (*M* = 0.93). Integrating advancements such as AI into these frameworks could further enhance their utility, enabling real-time, personalized predictions while facilitating inter-hospital comparability.

We believe that both tools, survival prediction and outcome comparison, are valuable in clinical practice. The Trauma Audit and Research Network (TARN), the largest trauma registry in Europe, employs survival probability for prediction and comparative outcome analysis through DEF statistics (*M*, *Z*) for inter-hospital comparability (60). These tools are invaluable for objectively addressing unfavorable outcomes, facilitating the identification of contributing factors to unexpected results, and improving the quality of trauma center care. Internal validation of Peds-TRISS confirmed strong performance across all metrics (Figure 3).

Given the nature of our patients' injuries and Peds-TRISS's performance metrics, it could serve as a local reference or be applied in similar contexts, underscoring the value of developing new models based on regional data (61, 62). Moreover, its applicability extends beyond Colombia, offering potential value for regions with similar epidemiological challenges, including countries across Latin America where violence-related injuries remain a substantial burden.

The critical burden of violence-related injuries in our region, disproportionately affecting children and adolescents, underscores the urgent need for tailored solutions in trauma care. Models like Peds-TRISS offer a starting point for addressing these challenges, providing a foundation for both clinical decision-making and broader public health strategies. Healthcare professionals and researchers must actively engage in developing and disseminating evidence-based tools that identify gaps, inform interventions, and ultimately reduce the societal impact of pediatric trauma. Collaborative efforts, supported by advancements in predictive technologies and an understanding of regional epidemiological patterns, are essential to creating sustainable solutions for improving outcomes in vulnerable populations.

### 4.1 Limitations, strengths and future directions

This study has limitations inherent to its retrospective, single-center design, which may restrict the generalizability and transportability of the results. External validation in other pediatric cohorts is therefore essential to assess its broader applicability. Furthermore, the high proportion of deaths due to violence among adolescents may limit the model's applicability in regions with different injury epidemiology. However, it is a current and real issue in Latin America, making the model potentially useful for similar geographical and epidemiological settings. Future multicenter and prospective studies would further support the validity and applicability of Peds-TRISS.

The strengths of our study include a large pediatric cohort, the first in Latin America to adapt and apply the TRISS model in children, providing valuable information on pediatric trauma care in Latin America, where trauma registries are limited, and local data are scarce.

## 5 Conclusions

In conclusion, this study revealed a high rate of violence-related deaths among adolescents, highlighting a critical public health challenge. While the discriminatory power of the Peds-TRISS model was nearly identical to that of the original TRISS, its superior calibration underscores its potential to more accurately align predicted and observed outcomes across varying levels of risk. Given the characteristics of our cohort and the results obtained, the model appears promising for similar contexts in Latin America. Further validation through prospective, multicenter studies is recommended to strengthen the evidence supporting its use and to inform the development of tailored and effective pediatric trauma care interventions

## Data Availability

The raw data supporting the conclusions of this article will be made available by the authors, without undue reservation.
